# Effects of targeted mild hypercapnia versus normocapnia on cerebral oxygen saturation in patients undergoing laparoscopic hepatectomy under low central venous pressure: a prospective, randomized controlled study

**DOI:** 10.1186/s12871-023-02220-y

**Published:** 2023-07-31

**Authors:** Huayan Lv, Chang Xiong, Bo Wu, Zhijian Lan, Duojia Xu, Dingnan Duan, Xiaoxia Huang, Jun Guo, Shian Yu

**Affiliations:** 1 Department of Anesthesiology, Jinhua Hospital Affiliated to Zhejiang University School of Medicine, Jinhua, Zhejiang Province People’s Republic of China; 2 Department of Hepatological Surgery, Jinhua Hospital Affiliated to Zhejiang University School of Medicine, Jinhua, Zhejiang Province People’s Republic of China

**Keywords:** Laparoscopic hepatectomy, Regional cerebral oxygen saturation, Ventilation

## Abstract

**Background:**

Laparoscopic hepatectomy under low central venous pressure (LCVP) is associated with intraoperative organ hypoperfusion, including cerebral hypoperfusion. We hypothesized that a ventilation strategy designed to achieve targeted mild hypercapnia (TMH) (end-tidal carbon dioxide partial pressure [PetCO_2_] of 45 ± 5 mmHg) rather than targeted normocapnia (TN) (PetCO_2_ of 30 ± 5 mmHg) would increase regional cerebral oxygen saturation (rSO_2_) during laparoscopic hepatectomy under LCVP.

**Methods:**

Eighty patients undergoing laparoscopic hepatectomy under LCVP were randomly divided into the TMH group (*n* = 40) and the TN group (*n* = 40). Mechanical ventilation was adjusted to maintain the PetCO_2_ within the relevant range. Cerebral oxygenation was monitored continuously using the FORE-SIGHT system before anesthetic induction until the patient left the operating room. Patient and surgical characteristics, rSO_2_, intraoperative hemodynamic parameters (CVP, mean artery blood pressure [MAP], and heart rate), PetCO_2_, intraoperative blood gas analysis results, and postoperative complications were recorded.

**Results:**

No significant differences were observed in CVP, MAP, and heart rate between the two groups during surgery. The rSO_2_ was significantly lower in the TN group on both the left and right sides during the intraoperative period (*P* < 0.05), while the TMH group had a stable rSO_2_. In the TN group, the mean rSO_2_ decreased most during liver parenchymal transection when compared with the baseline value (*P* < 0.05). The mean (standard deviation) percentage change in rSO_2_ from baseline to parenchymal transection was − 7.5% (4.8%) on the left and − 7.1% (4.6%) on the right. The two groups had a similar incidence of postoperative complications (*P* > 0.05).

**Conclusion:**

Our findings demonstrate that rSO_2_ is better maintained during laparoscopic hepatectomy under LCVP when patients are ventilated to a PetCO_2_ of 45 ± 5 mmHg (TMH) than a PetCO_2_ of 30 ± 5 mmHg (TN).

**Trial registration:**

ChiCTR2100051130(14/9/2021).

## Introduction

In patients undergoing laparoscopic hepatectomy, low central venous pressure (LCVP) is recommended to reduce the volume of blood loss and the surgical duration [1, 2]. The most common method used to achieve the LCVP during laparoscopic hepatectomy is fluid restriction before and during the transection phase, which, when combined with intraoperative blood loss, may result in hemodynamic instability and increase the risk of hypotension and organ hypoperfusion [3]. Relative hypotension and hypoperfusion may in turn cause organ dysfunction. A previous retrospective study showed that approximately 17% of patients developed postoperative biochemical acute kidney injury after liver resection under LCVP [3]. Another prospective observational study showed that approximately 30% of patients developed postoperative myocardial injury and transient acute kidney injury with liver resection under LCVP [4]. Therefore, anesthesiologists should pay careful attention to intraoperative organ hypoperfusion, the organ oxygen supply, and the consumption equilibrium when utilizing the LCVP technique during liver resection.

Previous study showed that the decrease in regional cerebral oxygen saturation (rSO_2_) measured by near-infrared reflectance spectroscopy (NIRS) during abdominal surgery was higher than initially thought [5], especially in older patients and patients with hypertension [5, 6]. However, few studies have examined the intraoperative rSO_2_ in patients under LCVP. Several physiologic alterations occur during laparoscopic hepatectomy under LCVP, including organ hypoperfusion during the LCVP phase, and the reverse Trendelenburg position also plays an important role. Such physiologic alterations may place the patient at risk of cerebral hypoperfusion.

During laparoscopic surgery, the most common gas involved in pneumoperitoneum to facilitate the surgical view is carbon dioxide (CO_2_), and hypercapnic acidosis is a common consequence of long surgical procedures. In the face of hypercapnia, some anesthesiologists adopt a do-nothing policy, while some are inclined to increase the tidal volume and respiratory rate for ventilation. However, the optimal method is uncertain. Nevertheless, it is known that hypercapnia increases cerebral blood volume through arterial vasodilation [7], and a recent study showed that mild hypercapnia is associated with a stable increase in the rSO_2_ from baseline compared with normocapnia [8]. Therefore, we hypothesized that mild hypercapnic acidosis may improve the rSO_2_ in patients undergoing laparoscopic hepatectomy under LCVP in the reverse Trendelenburg position and may enhance postoperative recovery.

In this randomized controlled trial, we examined cerebral rSO_2_ in patients undergoing laparoscopic hepatectomy under LCVP. We evaluated the efficacy and safety of targeted mild hypercapnia (TMH) (end-tidal carbon dioxide partial pressure [PetCO_2_] of 45 ± 5 mmHg) versus targeted normocapnia (TN) (PetCO_2_ of 30 ± 5 mmHg) during laparoscopic hepatectomy under LCVP.

## Methods

### Patients

This prospective randomized controlled trial was approved by the Medical Ethics Committee of Jinhua Hospital Affiliated to Zhejiang University on 15 December 2020. The trial was performed from October 2021 to November 2022. Written informed consent was obtained from all patients. The study has been registered in the Chinese Clinical Trial Registry (http://www.chictr.org.cn, ChiCTR2100051130).

Eighty consecutive patients who were classified as American Society of Anesthesiologists (ASA) grade I–II, aged 18–70 years, scheduled to undergo laparoscopic hepatectomy under LCVP for at least 2 h under general anesthesia, and operated on by a single surgeon were enrolled in this study. Participants with a body mass index (BMI) of > 35 kg/m^2^ or < 18 kg/m^2^, uncontrolled hypertension or orthostatic hypotension, severe heart diseases (including previous myocardial infarction, cardiac insufficiency, moderate to severe heart valve regurgitation, or severe arrhythmia), chronic obstructive pulmonary disease, hepatic failure, renal failure, neuropsychiatric diseases, a preoperative Mini-Mental State Examination score of < 24, or a history of stroke or brain trauma surgery were excluded.

All patients who met the inclusion criteria were documented consecutively. Randomization was performed after the patients were deemed eligible and had agreed to participate in the study. The computer-generated random numbers were sealed in opaque envelopes and kept by a nurse. The envelopes were opened after the patients had entered the operating room. The patients were randomly divided into the TMH group (*n* = 40) or the TN group *(n* = 40). The PetCO_2_ was maintained at 40–50 mmHg in the TMH group and at 25–35 mmHg in the TN group. The patients, the surgeons, and the researcher who collected the follow-up survey data and performed the statistical analysis were blinded to the group allocation. However, the attending anesthetist was not blinded to the group allocation and thus did not participate in the follow-up work.

### Anesthetic procedures

The anesthetic protocol was carefully standardized in all patients. Patients were not administered premedication or an intravenous line before entering the operation room. After the patients had entered the operating room, a peripheral intravenous line was established by the nurse. Then, radial arterial puncture and double-lumen right internal jugular catheterization were performed by the attending anesthetist under local anesthesia. Patients were then administered sufentanil (0.8 µg/kg), etomidate (0.3 mg/kg), and cis-atracurium (0.3 mg/kg) for induction of general anesthesia. General anesthesia was maintained with propofol, remifentanil, and cis-atracurium, as well as inhalational sevoflurane (0.5–1%). During anesthetic induction, the inhalational oxygen concentration was 100%, and a 50% oxygen-to-air mixture was used to maintain anesthesia. During surgery, the bispectral index score(BIS) of anesthetic depth was maintained between 40 and 60. Standard intraoperative monitoring included electrocardiography, pulse oximetry (SpO_2_), invasive mean arterial pressure (MAP), CVP, BIS, nasopharyngeal temperature, and PetCO_2_. The method to lower the CVP during laparoscopic hepatectomy has been used widely, and includes fluid restriction, patient positioning with the upward head tilt, and use of vasodilators or diuretics to achieve a CVP of < 5 cmH_2_O at the beginning of liver parenchymal transection [9]. If the CVP did not reach the target value, the patient remained in the study according to the intention-to-treat principle [10]. After completion of parenchymal transection, the rate of intravenous fluid administration was increased to maintain a normal intravascular volume. At the end of surgery, the patients woke up and were extubated in the operating room before being sent to the post-anesthesia care unit (PACU). During surgery, perioperative hypotension was defined as a 20% reduction of MAP from baseline. Phenylephrine (1–2 µg/kg) was administered if the MAP decreased by more than 20–30%. Packed red blood cells were transfused if the intraoperative red blood cell volume was < 25%.

### Ventilation management and PetCO_2_ measurement

After anesthetic induction and tracheal intubation, mechanical ventilation was performed with a fraction of inspired oxygen (FiO_2_) of 50% and a tidal volume at 8 mL/kg. The minute ventilation was adjusted to maintain the PetCO_2_ concentration between 25 and 35 mmHg (target partial pressure of CO_2_ in arterial blood [PaCO_2_] of 35 mmHg) in the TN group and between 40 and 50 mmHg (target PaCO_2_ of 50 mmHg) in the TMH group. The PetCO_2_ was monitored continuously during surgery and recorded automatically using a computer. In addition, blood samples were collected from the radial artery for arterial BGA (blood gas analyzer: GEM Premier 3500, Instrumentation Laboratory, Lexington, MA, US) at the beginning and end of liver parenchymal transection. In general, the PetCO_2_ can be lower than the PaCO_2_ by approximately 5 mmHg, as observed in our clinical practice. However, the PaCO_2_–PetCO2 gradient may not be maintained throughout surgery. Therefore, the respiratory parameters were adjusted based on the PetCO_2_ and PaCO_2_, and the PaCO_2_ was maintained at < 60 mmHg throughout the intraoperative period.

### Cerebral oxygenation measurements

After performing basic monitoring, non-invasive cerebral oxygenation sensors were applied bilaterally to both sides of the forehead (near the frontotemporal hairline and avoiding the frontal sinus) after cleansing the skin with facial scrub. Monitoring of cerebral oxygenation was initiated before the induction of general anesthesia. During surgery, cerebral oxygenation was monitored continuously using the FORE-SIGHT system (CAS Medical Systems, US) under the supervision of the attending anesthesiologist. The regional oximetry device used NIRS to measure the oxygen saturation of hemoglobin in the local brain tissue and displayed both absolute and trend rSO_2_ values on both sides of the brain. Only the absolute cerebral oxygenation data were extracted and analyzed. The cerebral oximetry and BIS sensors were fixed and covered with a surgical drape to prevent ambient light interference during surgery.

### Outcomes and perioperative data collection

The primary outcome was the absolute difference in rSO2 between the TMH and TN groups during surgery. The secondary endpoints were the effects of mild hypercapnia on the incidence of postoperative delirium, postoperative outcomes and perioperative complications, intraoperative pH, bicarbonate, base excess, serum potassium, and length of hospital stay (LOS).

On arrival at the operating room, the heart rate, SpO_2_, MAP, CVP, rSO_2_, and other indicators were recorded before the induction of general anesthesia. The initial values were obtained while breathing a 50% oxygen-to-air mixture via a face mask. These variables (heart rate, SpO_2_, MAP, CVP, BIS, rSO_2_, and PetCO_2_) were then automatically recorded by the computer and the FORE-SIGHT device every 5 min throughout surgery. The Case Report Form was manually completed by an anesthetic assistant according to the device data. After the induction of general anesthesia, pneumoperitoneum was created by skilled surgeons with CO_2_ gas at a pressure of 12–14 mmHg. After pneumoperitoneum had been established, the patient was positioned in the reverse Trendelenburg position (30° head high foot low) for the surgical procedure. Measurements were taken every minute, until 10 min after changing position. All of the data were collected until the patient left the operating room. After withdrawal of anesthesia at the end of surgery, the time required for tracheal extubation and the time required to leave the PACU were recorded. Blood samples were collected from the radial artery at least two times for BGA, and the pH, PaCO_2_, partial pressure of oxygen (PaO_2_), bicarbonate concentration, lactate concentration, potassium concentration, and hemoglobin concentration were recorded.

We divided the procedure into seven stages according to intraoperative fluid management and operative position, as follows: Phase 1: after entering the operating room and before the induction of general anesthesia; Phase 2: after the induction of general anesthesia and before cutting the skin; Phase 3: from cutting the skin to pneumoperitoneum establishment; Phase 4: after adjusting the position to the reverse Trendelenburg position and until 10 min after changing position; Phase 5: liver parenchymal transection; Phase 6: after parenchymal transection; Phase 7: after endotracheal tube extubation and before admission to the PACU.

In addition, the patients’ characteristics, including age, sex, BMI, ASA grade (I/II), and underlying diseases, were recorded. The surgical techniques used for hepatectomy were also recorded. Postoperative outcomes and perioperative complications, including nausea and vomiting, surgical site infection, intra-abdominal infection, biliary fistula, ileus, hypoxemia, prolonged mechanical ventilation (> 48 h), stroke, venous gas embolism, acute myocardial infarction, acute renal failure, and cardiac arrest, were also recorded. Postoperative delirium was assessed using the validated and widely used Confusion Assessment Method rating scale at around 24 h after surgery by one physician.

### Statistical analysis

The predefined primary endpoint was the cerebral oxygenation during laparoscopic hepatectomy under LCVP achieved by anesthetic intervention. In a previous study of patients undergoing major surgery, the mean (standard deviation [SD]) percentage change in rSO_2_ was 8.56% (18.90%) in the TMH group and − 5.48% (18.94%) in the TN group [8]. The Student’s t-test (two-tailed) was performed using the G-power program, which showed that 34 patients per group were required to detect a clinically relevant difference (ɑ = 0.05, type II error β = 0.2, effect size = 0.7). An intraoperative dropout rate of 15% was assumed. Therefore, the total sample size was 80 patients.

IBM SPSS statistical software, version 20 (IBM, Chicago, IL, US) was used for the statistical analysis. The distribution normality of continuous variables was measured using the Shapiro–Wilk test. Normally distributed continuous data were compared using the Student’s t-test, while non-normally distributed continuous data were compared using the Mann–Whitney U test. Continuous data are presented as the mean (SD) or median (interquartile range). The repeated-measures analysis of variance was used for within-group comparisons of the different time points. Categorical data were compared using the chi square test and are presented as number (percentage). A *P* value of < 0.05 was considered statistically significant for all analyses.

## Results

**Fig. 1:**
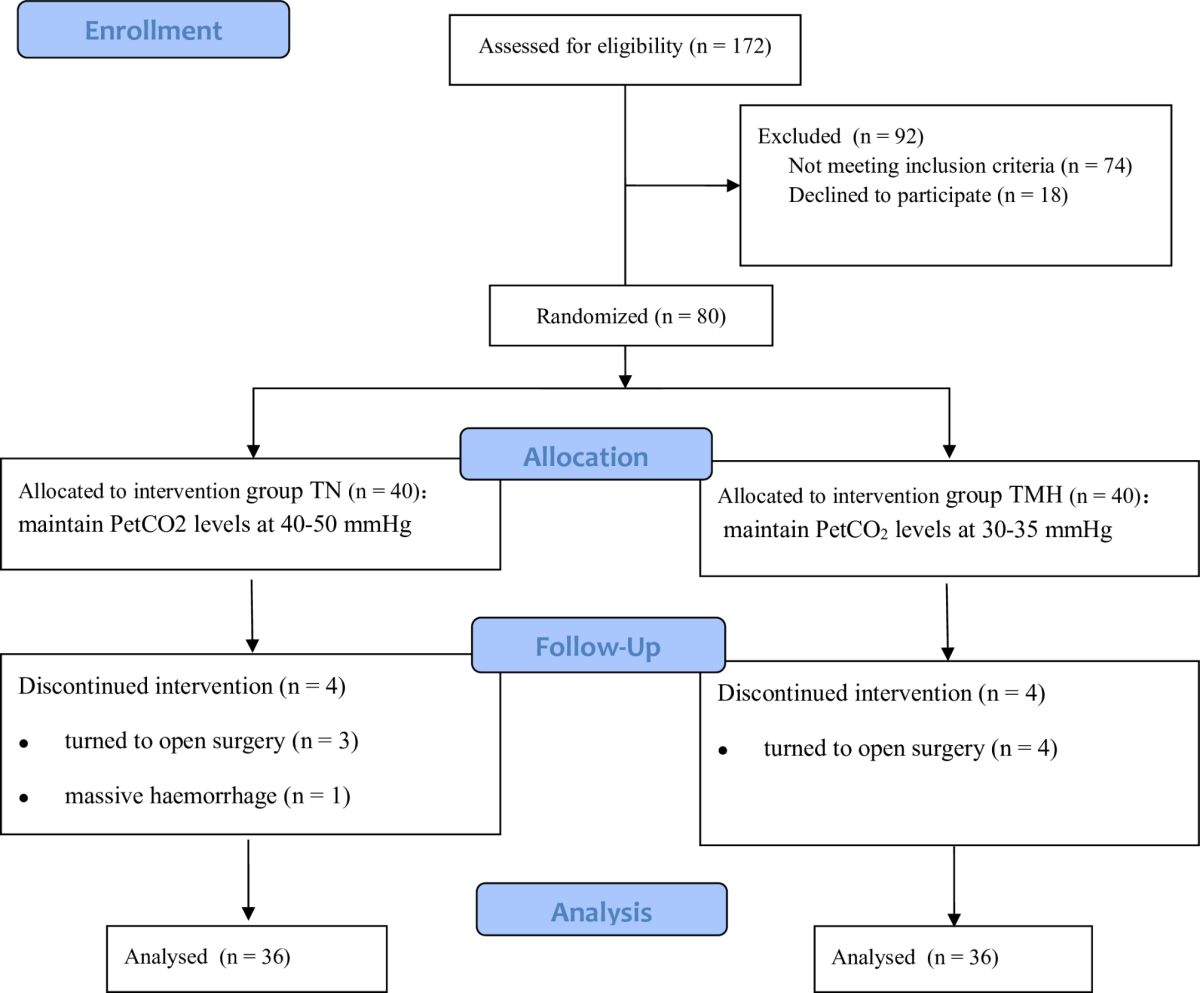
The CONSORT flow diagram

Among the 80 patients initially included in the study, seven patients (three in the TN group and four in the TMH group) had unexpected surgical difficulties and were converted to open surgery, and one patient in the TN group with sustained hypotension and intraoperative bleeding of > 1500 mL was excluded. Therefore, a total of 72 patients (90%) completed the study. The CONSORT flow diagram is shown in Fig. 1.

Patients’ characteristics, including sex, age, BMI, ASA grade, and underlying diseases, were not significantly different between the two groups (Table 1). In terms of surgery, there were no significant differences in the total duration of surgery, hepatic hilum occlusion, type of resection, rate of malignancy, total infused propofol, total intraoperative fluid administration, urine output, blood loss volume, or postoperative hemoglobin concentration between the two groups (Table 2).

**Table 1:** Patients Characteristics

	TN (n = 36)	TMH (n = 36)	*p*-value
Sex (male/female)	24/12	29/7	0.181
Age (years)	58.0 ± 10.2	55.5 ± 11.2	0.315
BMI (kg/m^2^)	22.2 ± 2.6	23.2 ± 2.5	0.102
ASA status (ǀ/ǁ)	8/28	10/26	0.586
Underlying diseases			
Hypertension	15	13	0.629
Diabetes mellitus	6	5	0.743
Liver cirrhosis	9	11	0.599
Pre-op Hb (g/dL)	13.1 ± 1.5	13.1 ± 1.6	0.885

Values are expressed as mean ± SD or number. *BMI* body mass index, *ASA* American Society of Anesthesiologists, *Pre-op Hb* preoperative hemoglobin

**Table 2:** Surgical characteristics

	TN (n = 36)	TMH (n = 36)	*p*-value
Duration of surgery (min)	238.2 ± 89.5	217.4 ± 82.1	0.308
Malignancy (%)	18(50.0%)	25(69.4%)	0.093
Pringle maneuvern (%)	25 (69.4%)	26 (72.2%)	0.795
Inflow occlusion time (min)	30 (0, 60)	30 (5, 45)	0.607
Resection types (n)			0.578
1 segment 2 segments Hemihepatic resection	9 18 9	13 16 7	
Total infused propofol(mg)	733.6 ± 299.6	743.1 ± 281.9	0.890
Fluid administered (mL)	2480.6 ± 825.3	2400.0 ± 874.4	0.689
Urine (mL)	498.6 ± 266.3	498.1 ± 247.7	0.993
Bleeding (mL)	315.8 ± 215.3	333.6 ± 260.8	0.753
Blood transfusion (%)	4(11.1%)	3(8.3%)	0.691
Post-op Hb (g/dL)	12.2 ± 1.3	12.3 ± 1.6	0.745

Values are expressed as mean ± SD, median (25th,75th) or number (%). *Post-op Hb* postoperative hemoglobin

**Fig. 2:**
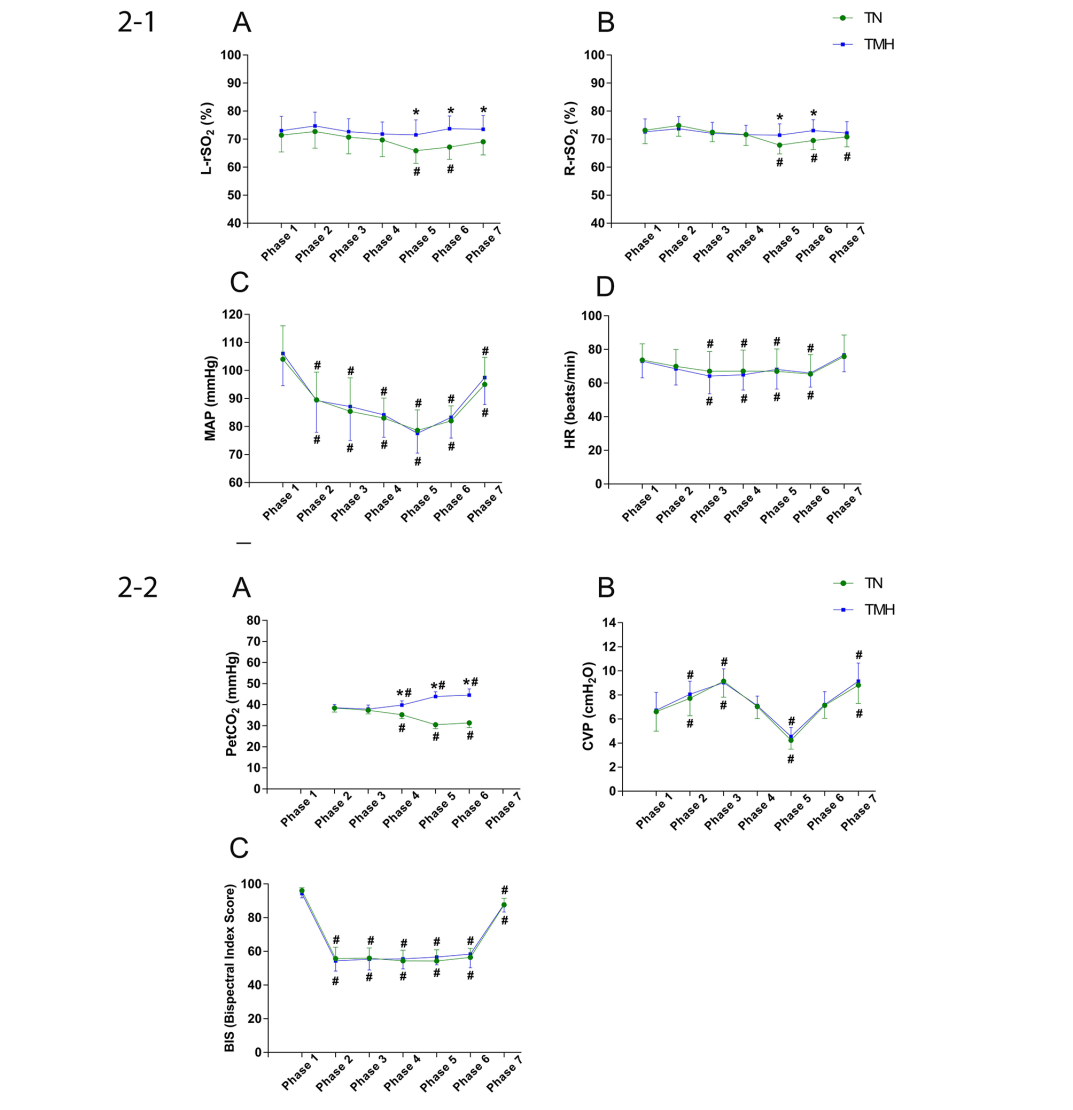
Changes from the Phase 1 until Phase 7. Figures 2–1 Changes in (**a**) left regional cerebral oxygen saturation (L-rSO_2_), (**b**) right regional cerebral oxygen saturation (R-rSO_2_), (**c**) mean arterial pressure (MAP), (**d**) heart rate (HR). Figure 2–2 (**a**) end-tidal carbon dioxide partial pressure (PetCO_2_), (**b**) central venous pressure (CVP) and (**c**) bispectral index score(BIS). Phase 1 = baseline before anesthesia induction; Phase 2 = post-induction; Phase 3 = post cutting the skin; Phase 4 = after the reverse Trendelenburg position; Phase 5 = parenchymal transection; Phase 6 = after parenchymal transection, fluid resuscitation; Phase 7: after endotracheal tube extubation. Groups: TN = targeted normocapnia; TMH = targeted mild hypercapnia. Data are expressed as mean ± standard deviation. *p < 0.05 compared between two groups; #P < 0.05 vs. Baseline in each group

The intraoperative parameters in both groups are shown in Figs. 2–1 and 2–2. The mean rSO_2_ was greater in the TMH group than in the TN group during and after parenchymal transection (Phase 5 and Phase 6, Figs. 2–1a and b). The mean rSO_2_ decreased most during parenchymal transection stage (Phase 5) in both groups(Fig. 2–1a and b). The mean rSO_2_ and the minimum rSO_2_ in Phase 5 was lower in the TN group compared with the TMH group on both left and right hemispheres (P < 0.05)(Table 3). On both sides, the decline in rSO_2_ (%ΔrSO_2_) was greater in the TN group than in the TMH group during Phase 5 (*P* < 0.05)(Table 3). In terms of the PetCO_2_, the mean value was significantly lower during Phase 4, Phase 5, and Phase 6 in the TN group than in the TMH group (*P* < 0.001)(Fig. 2–2a). The MAP, heart rate, CVP and BIS did not differ significantly throughout the measurement period between the two groups(Fig. 2–1c, d, Fig. 2–2b, c).

**Table 3:** rSO_2_ values at baseline and parenchymal transection (phase 5)

	TN(n = 36)	TMH (n = 36)	*p*-value
**Left**			
Baseline	71.4 ± 6.0	73.0 ± 5.2	0.235
Mean rSO_2_ of phase 5	65.9 ± 4.5	71.5 ± 5.4	0.000
Min rSO_2_ of phase 5	62.3 ± 5.3	67.3 ± 7.4	0.002
Percentage change in Mean rSO_2_ of phase 5 from baseline (%ΔrSO_2_ )	-7.5 ± 4.8	-1.8 ± 7.0	0.000
Percentage change in Min rSO_2_ of phase 5 from baseline (%ΔrSO_2_ )	-12.6 ± 6.6	-7.7 ± 9.7	0.015
**Right**			
Baseline	73.2 ± 4.8	72.6 ± 4.6	0.617
Mean rSO_2_ of phase 5	67.9 ± 3.2	71.5 ± 4.1	0.000
Min rSO_2_ of phase 5	64.0 ± 4.1	67.4 ± 5.3	0.003
Percentage change in Mean rSO_2_ of phase 5 from baseline (%ΔrSO_2_ )	-7.1 ± 4.6	-1.4 ± 5.4	0.000
Percentage change in Min rSO_2_ of phase 5 from baseline (%ΔrSO_2_ )	-12.4 ± 6.0	-7.0 ± 7.2	0.001

Values are expressed as mean ± SD. *rSO*_*2*_ = regional cerebral oxygen saturation

The MAP decreased from initial values in both groups over time, the mean MAP decreased most during parenchymal transection stage (Phase 5) in both groups(Fig. 2–1c).Hypotension was present in 30 out of 36 participants in the TN group and 31 out of 36 participants in the TMH group. The incidence of interventions for MAP decreases was similar between groups(Table 4). The incidence of postoperative delirium (16.7% in the TN group and 11.1% in the TMH group, *P* = 0.496) was also similar between the two groups at 24 h after surgery. All the other recovery variables didn’t show any statistical difference between the two groups (*P* > 0.05)(Table 4). In terms of the arterial BGA (Table 5), the PaO_2_ was similar between the two groups. The mean PaCO_2_ was significantly higher in the TMH group (50.4 (44.8–58.7) mmHg) than in the TN group (38.3 (31.5–47.6) mmHg) (*P* < 0.001). The mean PetCO_2_, which was measured at the same time as the PaO_2_ and PaCO_2_, was significantly higher in the TMH group than in the TN group (41.9 vs. 31.2 mmHg, *P* < 0.001). The pH value was significantly lower in the TMH group than in the TN group (7.31 vs. 7.41, *P* < 0.001).

**Table 4:** Perioperative variables and postoperative recovery

	TN (n = 36)	TMH (n = 36)	*p*-value
Interventions for MAP decreases	30	31	0.743
Time to tracheal extubation	16.2 ± 3.9	14.8 ± 3.5	0.101
Time of PACU	54.4 ± 12.0	52.1 ± 11.5	0.398
Nausea	8	6	0.551
Vomiting	3	3	1
Surgical site infection	2	1	0.555
Intra-abdominal infection	1	0	0.314
Postoperative delirium	6	4	0.496
Length of stay (LOS)	9.0 ± 3.7	8.3 ± 3.6	0.423

Values are expressed as mean ± SD or number (%). *MAP =* mean arterial pressure, *PACU =* the post-anesthesia care unit

**Table 5:** Arterial blood gas values and the corresponding PetCO_2_

	TN (n = 36)	TMH (n = 36)	*p*-value
PH	7.41 ± 0.04	7.31 ± 0.04	0.000
PaO_2_ (mm Hg)	194.6 ± 35.0	186.3 ± 38.4	0.344
PaCO_2_ (mm Hg)	38.3 ± 4.0	50.4 ± 3.1	0.000
PetCO_2_ (mm Hg)	31.2 ± 2.3	41.9 ± 2.1	0.000
Bicarbonate (mmol/L)	24.2 ± 1.5	26.0 ± 1.7	0.000
Potassium (mmol/L)	3.6 ± 0.4	4.0 ± 0.4	0.001
Lactic acid	1.2 ± 0.3	1.4 ± 0.4	0.181

Values are expressed as mean ± SD. *PH* = potential of hydrogen. *PaO*_*2*_ = partial

pressure of oxygen in arterial blood. PaCO_2_ = partial pressure of carbon dioxide in arterial blood. PetCO_2_ = the end-tidal carbon dioxide partial pressure

## Discussion

In this prospective, single-center, single-blind, randomized controlled trial, we explored the effects of different PetCO_2_ values on the rSO_2_ in patients undergoing laparoscopic hepatectomy under LCVP. The TN group demonstrated an obvious decrease in rSO_2_ on both the left and right sides during surgery when compared with the baseline values (*P* < 0.05), while the TMH group demonstrated a stable rSO_2_. Therefore, during laparoscopic hepatectomy under LCVP, the rSO_2_ was better maintained under mild hypercapnia. However, the two groups had similar incidences of postoperative delirium and perioperative complications (*P* > 0.05).

Theoretically, elevated PaCO_2_ increases cerebral blood flow, independent of autoregulation [7, 11, 12]. However, virtual changes in rSO_2_ as a result of changes in cerebral blood flow can be affected by hemoglobin concentration, FiO_2_, MAP, position, cerebral metabolic rate, oxygen dissociation curve, and ratio of cerebral arterial to venous blood volume, which can fluctuate during surgery. A previous study showed that cerebral oxygenation may not precisely reflect a decrease in cerebral blood flow during mild hypergravity [13]. Although NIRS does not directly measure cerebral blood flow, many studies have addressed the positive correlation between rSO_2_ and PetCO_2_ [8, 14]. In our study, influencing variables, such as the FiO_2_, hemoglobin concentration, MAP, BIS, method of anesthesia, and intraoperative position, were similar between the TMH and TN groups. We also observed that a higher rSO_2_ was associated with an elevated PaCO_2_. This is in agreement with a previous study showing the same finding in major surgery performed in different positions [8], as well as in shoulder surgery with the patient in the beach chair position [14] and in patients with morbid obesity undergoing laparoscopic bariatric surgery in the reverse Trendelenburg position [15].

Despite the use of a protocol designed to optimize MAP in the present study, hypotension was a frequent occurrence. In 84.7% of the patients (30 in the TN group and 31 in the TMH group), the MAP decreased by more than 20–30%, which required intervention. Intraoperative blood loss is challenging for hepatic surgeons due to the complicated anatomical structure and double blood supply system of the liver, and it is one of the major factors determining the success of laparoscopic surgery. Many reports have suggested an association between a CVP of < 5 mmHg and less blood loss or less transfusion during hepatectomy, so the LCVP technique is recommended in liver resection surgery to reduce bleeding [2, 16, 17]. Conventional LCVP methods include intravenous fluid restriction, an increased anesthetic depth, and the use of vasodilators (such as glyceryl trinitrate) and diuretics [17, 18]. A worldwide survey on liver resection reflected that the laparoscopic approach is widely used globally. To reduce the volume of intraoperative blood loss, 88% of centers restricted intraoperative fluid infusion to reduce the CVP [19]. However, other studies have reported that restricting intraoperative fluids can lead to hemodynamic instability that requires additional vasopressors [2, 20]. Although LCVP has been recommended for hepatectomy for many years, its widespread use is still limited [18]. The LCVP strategy is associated with several problems that add to the workload of anesthesiologists. In particular, there is concern about the possibility of relative hypotension and organ hypoperfusion, which can lead to morbidity.

The present study showed that the TMH group had a stable rSO_2_ during surgery, while other studies have shown that mild hypercapnia is associated with an increase in the rSO_2_ from baseline compared with normocapnia [8, 21]. The discrepancy between these reports and our results might be explained by the intravascular blood volume difference. In the present study, less than 0.5 L/hour of fluid was administered to achieve a CVP of < 5 cmH_2_O during parenchymal transection, which is insufficient to keep the hemodynamics steady in anesthetized patients. Changes in hemodynamics may influence cerebral blood flow. Murphy and his colleagues found that a decrease in rSO_2_ of 20% was associated with hypotension (a decrease of ≥ 20% in MAP from baseline) [14]. Similarly, a positive correlation between MAP and rSO_2_ was observed by Jing et al. [22]. Schramm and his colleagues demonstrated that administering more fluids to maintain a constant cardiac output (CO) and MAP during neurosurgery in the sitting position could maintain the rSO_2_ within the normal range. Moreover, the rSO_2_ correlated more strongly with CO (flow) than with MAP (pressure) [23]. In patients undergoing laparoscopic hepatectomy, the LCVP technique may lead to hypovolemia. Hypovolemia combined with the vasodilator effect of anesthesia induces an inadequate cardiac preload and CO [20]. In the present study, we did not monitor the CO; however, we did observe a decrease in the MAP of > 20% in 84.7% of patients when the LCVP technique was adopted. We used phenylephrine to keep the MAP within the predefined limits. A previous study found that prophylactic phenylephrine infusion after spinal anesthesia during cesarean delivery was associated with a significant decrease in rSO_2_ [24]. These factors may have attenuated the effects of hypercapnia on the increase in cerebral blood flow. Therefore, one possible explanation for the lack of an increase in rSO_2_ in the present study, which contrasts with the observed increase under mild hypercapnia, is hypotension with possible coexistence of a low CO attributable to the LCVP. Furthermore, phenylephrine may also play an important role.

Several studies have shown that body position can influence rSO_2_. Some clinical trials have demonstrated that patients in the beach chair position have significantly lower rSO_2_ values than patients in the supine position during shoulder surgery under general anesthesia [25, 26]. Mol et al. reported that rSO_2_ decreased after standing up from the supine and sitting positions in healthy individuals [27]. Lee et al. reported that rSO_2_ decreased in association with the Trendelenburg position and pneumoperitoneum during laparoscopic surgery [28]. While, Karaveli et al. found that the rSO_2_ increased significantly during pneumoperitoneum combined with the Trendelenburg position compared with the supine position [29]. Moreover, a prospective clinical trial found that the rSO_2_ increased slightly in patients undergoing orthopedic surgery and in awake volunteers in the prone position [30]. In the present study, the influence of a change in position from the supine position to the reverse Trendelenburg position on rSO_2_ was examined, but no significant decrease in rSO_2_ was observed. This result is similar to to Jo’s study [31]. To our knowledge, studies examining the influence of the reverse Trendelenburg position on cerebral oxygenation in anesthetized surgical patients are scarce.

In the present study, the incidence of postoperative delirium after surgery was similar between the two groups. We speculate that this may have occurred for the following reasons. First, we excluded patients aged > 70 years who are at a higher risk of cognitive dysfunction. Second, our study was performed at a setting with a well-implemented Enhanced Recovery After Surgery protocol, which may have accelerated patient recovery. Third, 10 mg dexamethasone was administered to every patient after the induction of general anesthesia at our center, which may have reduced the occurrence of postoperative delirium [32]. Fourthly, maybe the difference in rSO2 detected between groups in our study is of no clinically meaningful magnitude. Postoperative delirium is associated with increased mortality. Its incidence is impacted by multiple factors, and the association between cerebral desaturation and postoperative delirium is still controversial [8, 31]. The conventional approach that is used to explore the correlation between postoperative delirium and rSO_2_ is the linear correlation, which mostly focuses on the mean and lowest rSO_2_ values and may ignore the complexity of the brain. Wang et al. introduced a novel non-linear measure with approximate entropy and sample entropy to explore the relationship between the intraoperative rSO_2_ and postoperative delirium, which may offer new insights for clinical research [33]. Further similar studies are required to answer the question of whether there is an association between cerebral hypoxia and postoperative delirium.

Inconsistent with a previous study [8], we found that the PaCO_2_–PetCO_2_ gradient was not maintained throughout surgery. The PaCO_2_–PetCO_2_ gradient increased along with prolongation of pneumoperitoneum. This phenomenon has been reported previously in laparoscopic colon surgery [34]. In general, PetCO_2_ can be synchronized to accurately reflect the change in PaCO_2_, with the assumption that the physiological dead space and intrapulmonary shunt volume remain constant [35]. However, CO_2_ pneumoperitoneum can increase the physiological dead space. As surgery continues, vast amounts of CO_2_ are absorbed through the peritoneum, and to achieve the desired PaCO_2_ target range, the minute ventilation is further increased, which increases the pleural pressure and the intrapulmonary shunt volume. Consequently, changes in the physiological dead space and intrapulmonary shunt volume result in less exhaled CO_2_ and an increase in the PaCO_2_–PetCO_2_ gradient.

The present study has several limitations that should be noted. First, the study was conducted at a single center and was a single-blinded study. However, the attending anesthesiologist was inevitably aware of the group allocation and the measured intraoperative variables. Second, the risk of air embolism may increase in patients undergoing laparoscopic hepatectomy under LCVP. PetCO_2_ monitoring is an important method for early detection of air embolism. The PetCO_2_ suddenly increased in some patients during surgery, but we did not use intraoperative color Doppler ultrasound to make a definite diagnosis of air embolism in real-time. Therefore, air embolism was difficult to diagnose in the present study. Furthermore, air embolism can affect the data on brain rSO_2_. However, no cases of severe air embolism occurred in this study. Third, although LCVP and normal CO can co-exist with delicate anesthetic management, hemodynamic instability often occurs in patients with LCVP, possibly due to a low preload resulting from fluid restriction. However, we did not monitor the CO of the patients in this study. Finally, we only measured PaCO2 at the beginning and end of parenchymal transection but not at each stage in this study. We found an increase in the PaCO2–PetCO2 gradient intraoperatively, which may have caused some bias. Given these limitations, further well-designed, multi-center, randomized studies are needed to validate and establish the clinical significance of our findings.

## Conclusion

The results of the present study indicate that TMH contributes to the maintenance of rSO_2_ during laparoscopic hepatectomy under LCVP. An obvious decrease in the rSO_2_ was observed in the TN group when the LCVP technique was adopted. Therefore, clinicians should remain cautious about the application of LCVP, and meticulous care should be paid to control ventilation and hemodynamics, and to avoid hyperventilation.

## Data Availability

The datasets used and analysed during the current study are available from the corresponding author on reasonable request.

## Abbreviations

LCVP: Low central venous pressure
CVP: Low central venous pressure
rSO_2_: Regional cerebral oxygen saturation
TMH: Targeted mild hypercapnia
TN: Targeted normocapnia
PetCO_2_: End-tidal carbon dioxide partial pressure
MAP: Mean artery pressure
NIRS: Near-infrared reflectance spectroscopy
CO_2_: Carbon dioxide
ASA: American Society of Anesthesiologists
BMI: Body mass index
BIS: The bispectral index
(PACU: The post-anesthesia care unit
FiO_2_: Inspired oxygen
PaCO_2_: Target partial pressure of CO_2_ in arterial blood
BGA: Blood gas analyzer
PaO_2_: Partial pressure of oxygen
CO: Cardiac output
